# Mallards and Highly Pathogenic Avian Influenza Ancestral Viruses, Northern Europe

**DOI:** 10.3201/eid1110.050546

**Published:** 2005-10

**Authors:** Vincent J. Munster, Anders Wallensten, Chantal Baas, Guus F. Rimmelzwaan, Martin Schutten, Björn Olsen, Albert D.M.E. Osterhaus, Ron A.M. Fouchier

**Affiliations:** * Erasmus Medical Center, Rotterdam, the Netherlands; † Smedby Health Center, Kalmar, Sweden; ‡ Linköping University, Linköping, Sweden; § Umea University, Umea, Sweden; ¶ Kalmar University, Kalmar, Sweden

**Keywords:** avian influenza, influenza A virus, surveillance, wildlife, HPAI, pandemic, research

## Abstract

Outbreaks of highly pathogenic avian influenza (HPAI), which originate in poultry upon transmission of low pathogenic viruses from wild birds, have occurred relatively frequently in the last decade. During our ongoing surveillance studies in wild birds, we isolated several influenza A viruses of hemagglutinin subtype H5 and H7 that contain various neuraminidase subtypes. For each of the recorded H5 and H7 HPAI outbreaks in Europe since 1997, our collection contained closely related virus isolates recovered from wild birds, as determined by sequencing and phylogenetic analyses of the hemagglutinin gene and antigenic characterization of the hemagglutinin glycoprotein. The minor genetic and antigenic diversity between the viruses recovered from wild birds and those causing HPAI outbreaks indicates that influenza A virus surveillance studies in wild birds can help generate prototypic vaccine candidates and design and evaluate diagnostic tests, before outbreaks occur in animals and humans.

## Abstract 2

Surveillance studies in wild birds help generate prototypic vaccine candidates and diagnostic tests.

Wild birds, predominantly ducks, geese, and shorebirds, form the reservoir of influenza A viruses in nature (*1**,**2*). Influenza A viruses are subtyped on the basis of the antigenic properties of the hemagglutinin (HA) and neuraminidase (NA) glycoproteins, expressed on the surface of virus particles. To date, 16 HA and 9 NA subtypes have been detected in wild birds and poultry throughout the world (*3**,**4*). Viruses containing HA of subtypes H5 and H7 may become highly pathogenic after introduction in poultry and cause outbreaks of highly pathogenic avian influenza (HPAI, formerly termed fowl plague) (*1**,**2*). The switch from a low pathogenic avian influenza (LPAI) phenotype, common in wild birds and poultry, to the HPAI phenotype is achieved by the introduction of basic amino acid residues into the HA0 cleavage site (*5*). HPAI isolates have been obtained primarily from commercially raised birds such as chickens, turkeys, quail, guinea fowl, and ostriches (*6*). In the last decade, the frequency of detected HPAI outbreaks has increased, with outbreaks of avian influenza A viruses of subtype H5N2 in Mexico (1994); Italy (1997) and Texas (2004); H5N1 in Hong Kong (1997) and Southeast Asia (ongoing since 1997); H7N3 in Australia and Pakistan (1994); H7N4 in Australia (1997); H7N1 in Italy (1999); H7N3 in Chile (2002) and Canada (2003); and H7N7 in the Netherlands (2003) (*7**–**15*).

Influenza A viruses of subtypes H5 and H7 have been frequently detected in mammals (*16*). H7N7 viruses have been endemic in horses for some time (*17*), were transmitted from seals to humans in the United States in 1980 (*18**,**19*), and were isolated from humans in the United Kingdom in 1996 (*20*) and the Netherlands in 2003 (*12**,**13*). H7N2 and H7N3 influenza A viruses were isolated from humans in the United States in 2003 (*21**,**22*) and Canada in 2004 (*23*), respectively. HPAI H5N1 viruses circulating in Southeast Asia since 2003 have been detected in at least 108 human cases of respiratory illness, of which 54 were fatal (*24*). In addition, these H5N1 influenza A viruses have been detected in pigs (*25*), cats, leopards, and tigers (*26**–**29*) in Southeast Asia. As a consequence of the relatively frequent zoonoses caused by influenza A viruses of subtypes H5 and H7, these virus subtypes are given high priority with respect to pandemic preparedness.

Wild birds harbor the LPAI ancestral viruses of HPAI strains of poultry (and mammals). In our influenza A virus surveillance studies in wild birds in northern Europe, we detected numerous influenza A viruses of subtype H5 and H7 in Mallards (*Anas platyrhynchos*). We show that for each of the HPAI outbreaks that occurred in Europe since 1997, we have found close LPAI relatives in Mallards. Our observations indicate that influenza A virus surveillance in wild birds provides opportunities for pandemic preparation; the prototype influenza A viruses obtained from wild birds may guide production of vaccines as well as reagents to develop and validate diagnostic tests.

## Materials and Methods

### Specimens

**Figure 1:**
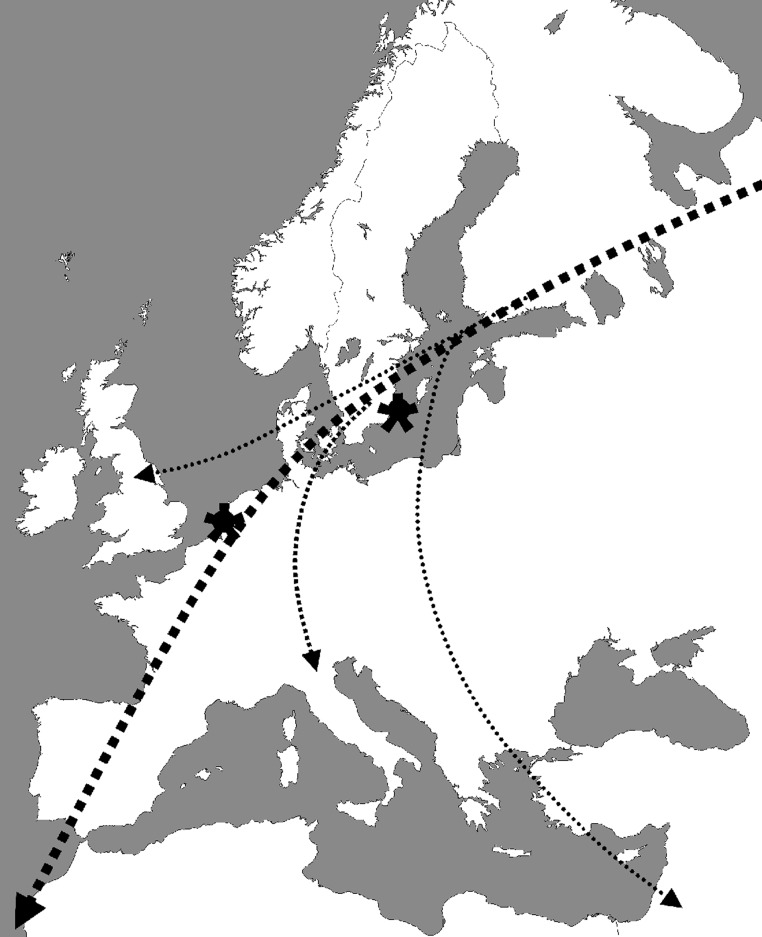
Main fall migration route of wild waterfowl in northern Europe (*31*). The sample locations Öland (Sweden) and Lekkerkerk and Krimpen a/d Lek (the Netherlands) are marked with asterisks.

In our ongoing influenza A virus surveillance studies in wild birds in northern Europe (*30*), Mallards were trapped with duck traps in Lekkerkerk and Krimpen aan de Lek in the Netherlands and Ottenby Bird Observatory on the southernmost point of the island Öland in Sweden (Figure 1). Cloacal samples were collected with cotton swabs and stored in transport media consisting of Hanks' balanced salt solution, 10% vol/vol glycerol, 200 U/mL penicillin, 200 μg/mL streptomycin, 100 U/mL polymyxin B sulfate, and 250 μg/mL gentamicin (MP Biomedicals, Zoetermeer, the Netherlands) at –70°C.

### RNA Isolation and Virus Detection

RNA isolation and reverse transcription–polymerase chain reaction (RT-PCR) were performed as described previously (*32*) for samples obtained until 2002. Beginning in 2003, RNA was isolated by using a MagnaPure LC system with the MagnaPure LC Total nucleic acid isolation kit (Roche Diagnostics, Almere, the Netherlands), and influenza A virus was detected by using a real-time RT-PCR assay (*33*). To ensure efficient influenza A virus detection, the published probe sequence was changed to 6-FAM-TTT-GTG-TTC-ACG-CTC-ACC-GTG-CC-TAMRA-3´, based on the avian influenza A virus sequences available from public databases. Amplification and detection were performed on an ABI7700 machine with the TaqMan EZ RT-PCR Core Reagents kit (Applied Biosystems, Nieuwerkerk aan den IJssel, the Netherlands) by using 20 μL eluate in an end volume of 50 μL. Pools of 5 individual samples were prepared and processed in parallel with several negative and positive control samples in each run. Upon identification of influenza A virus–positive pools, RNA isolation and RT-PCR procedures were repeated for the individual samples within each positive pool; individual RT-PCR–positive samples were subsequently used to isolate virus.

### Virus Isolation and Characterization

For influenza A virus RT-PCR–positive samples, 200 μL original material was injected into the allantoic cavity of 11-day-old embryonated hens' eggs. The allantoic fluid was harvested 2 days after injection, and influenza A virus was detected by using hemagglutination assays with turkey erythrocytes. When no influenza A virus was detected on the initial virus isolation attempt, the allantoic fluid was passaged once more in embryonated hens' eggs. Virus isolates were characterized with a hemagglutination inhibition (HI) assay with turkey erythrocytes and subtype-specific hyperimmune rabbit antisera raised against all HA subtypes (*4**,**34*).

### Sequence Analysis and Phylogenetic Trees

NA subtypes of influenza A virus isolates were characterized by RT-PCR and sequencing. RT-PCR and sequencing of the HA and NA genes were performed essentially as described by others (*35*). PCR products were purified from agarose gels with the Qiaquick Gel Extraction kit (Qiagen, Leusden, the Netherlands) and sequenced with the Big Dye terminator sequencing kit version 3.0 (Amersham Pharmacia Biotech, Roosendaal, the Netherlands) and an ABI PRISM 3100 genetic analyzer (Applied BioSystems), according to the instructions of the manufacturer. All primer sequences are available upon request. Nucleotide and amino acid sequences were aligned by using the ClustalW program running within the BioEdit software package, version 5.0.9 (*36*). We first generated trees for H5 and H7 by using all full-length HA1 amino acid sequences available from public databases. Amino acid sequence alignments were bootstrapped 100 times, and distance matrices were generated by using Kimura parameters. The trees were generated by using the UPGMA (unweighted pair-group method with arithmetic mean) clustering method of the Neighbor program of PHYLIP version 3.6 (*37*). The consensus of 100 UPGMA trees was calculated, and the branch lengths of this consensus tree were recalculated by using the Fitch program of PHYLIP 3.6.

For selected influenza A virus isolates of European origin, DNA maximum-likelihood trees were generated by using full-length HA nucleotide sequences from which the sequences encoding the HA cleavage site were excluded. Alignments were bootstrapped 100 times by using the Seqboot package of PHYLIP version 3.6, and trees were constructed with the Dnaml package, using 3 jumbles. The consensus tree was calculated by using the Consense package of PHYLIP 3.6; this tree was used as usertree in Dnaml to recalculate the branch lengths from the nucleotide sequences. Finally, the trees were rerooted at midpoint by using the Retree software of PHYLIP 3.6. Trees were visualized with the Treeview 1.6.6 program distributed with BioEdit version 5.0.9. All nucleotide sequences presented here are available from GenBank under accession numbers AY684894, AY338460, AY995883–AY995898, and AY999977–AY999991.

### Serology

HI assays were performed to compare the antigenic properties of influenza A virus strains by using postinfection ferret antisera and hyperimmune rabbit antiserum generated against the following influenza viruses: A/Tern/South Africa/61 (H5N3), A/Duck/Hong Kong/205/77 (H5N3), A/Hong Kong/156/97 (H5N1), A/Equine/Prague/1/54 (H7N7), A/Seal/Massachusetts/1/80 (H7N7), A/Mallard/Netherlands/12/00 (H7N3), A/Netherlands/033/03 (H7N7), and A/Netherlands/219/03 (H7N7), as described previously (*4**,**34*). HI assays were performed in duplicate. All serum samples were treated overnight with receptor-destroying enzyme at 37°C and subsequently incubated at 56°C for 1 hour. Twofold serial dilutions of each antiserum, starting at a 1:20 dilution, were tested for their ability to inhibit the agglutination of horse erythrocytes by 4 hemagglutinating units of influenza A virus. Serum dilutions were made in phosphate-buffered saline (PBS) containing 0.5% vol/vol bovine serum albumin (BSA, fraction V, Gibco, Breda, the Netherlands). Horse erythrocytes were stored in PBS containing 0.5% vol/vol BSA. In the HI assay, 50 μL of a 1% vol/vol horse erythrocyte dilution was added to each well (*38*).

## Results

### Avian Influenza A Virus in Wild Birds in Europe

Of 172 virus isolates obtained within this study period, 33 contained HA genes of subtypes H5 or H7, 6 were of subtype H5N2, 2 were H5N3, 1 was H5N6, 8 were H5N9, 1 was H7N3, 14 were H7N7, and 1 was H7N9. All H5 and H7 influenza A viruses were isolated from samples collected from Mallards during fall migration at marshalling sites in the Netherlands (1 H5 isolate from October 1999 and 1 H7 isolate from December 2000) and Sweden (all other H5 and H7 isolates collected from September to January 2002) (Figure 1).

### Characterization of H7 Influenza A Viruses

**Figure 2:**
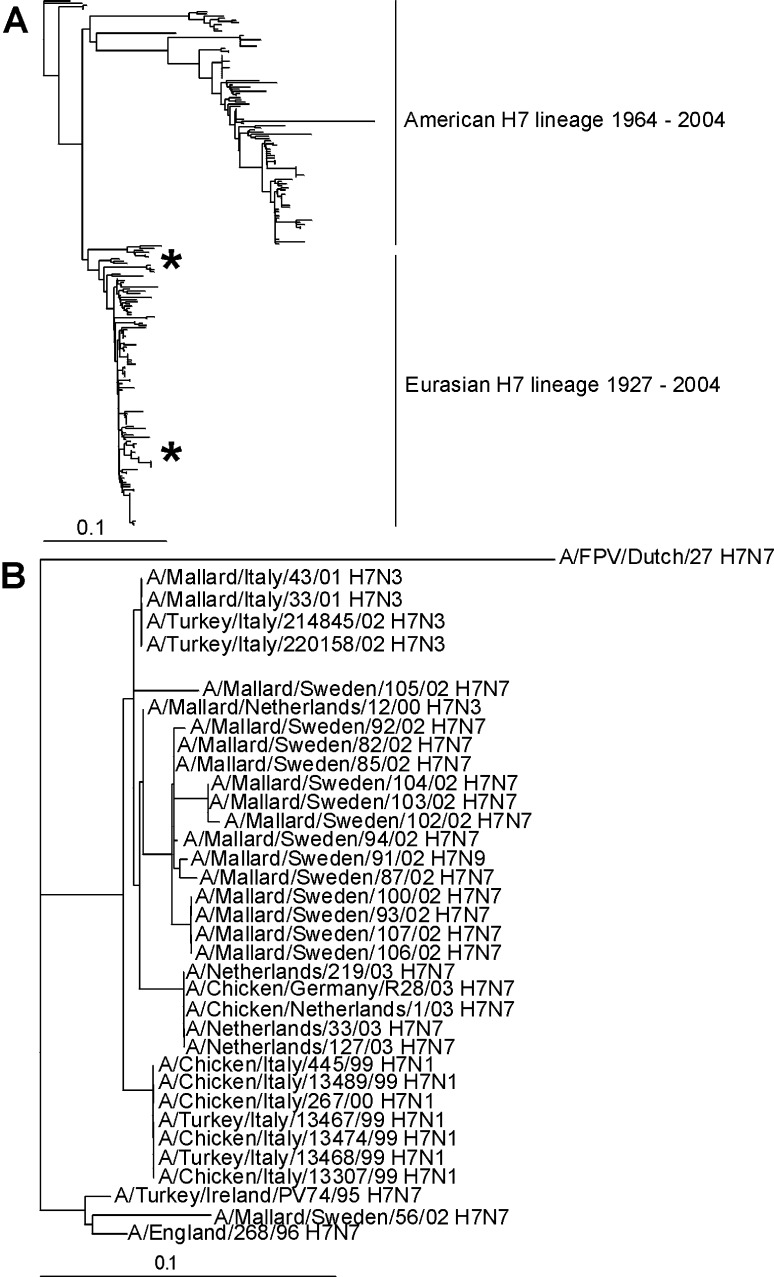
Phylogenetic trees of hemagglutinin H7 sequences. A) Phylogenetic tree based on the amino acid sequence distance matrix for the HA1 open reading frames of all H7 sequences available from public databases. The scale bar represents ≈10% of amino acid changes between close relatives. *Represents the locations of the Mallard influenza A virus isolates. B) DNA maximum likelihood tree for the European highly pathogenic avian influenza viruses and the low pathogenic avian influenza H7 influenza A viruses isolated from migrating Mallards by using A/FPV/Dutch/27 as outgroup. The scale bar represents 10% of nucleotide changes between close relatives.

Sequence analyses of the HA open reading frames (ORFs) of the 16 H7 influenza A viruses isolated from Mallards showed that the HA0 cleavage site lacked basic amino acid residues, which is typical for LPAI viruses. We next determined the genetic relationship between the HA genes of our H7 influenza A viruses isolated from European Mallards and those available from public sequence databases. The phylogenetic tree, based on HA1 amino acid sequences, showed the typical separation of H7 strains in the Eurasian and American genetic lineages. Within the Eurasian H7 HA lineage, the European Mallard influenza A viruses were found in different parts of the tree, clustering closely with influenza A viruses responsible for recent H7 HPAI outbreaks in Europe (Figure 2A). We next generated a DNA maximum-likelihood phylogenetic tree by using prototypic European Mallard influenza A viruses and strains representing each of the H7 HPAI outbreaks that occurred in Europe (H7N1 in Italy 2000/2001 and H7N7 in the Netherlands 2003) in the last decade (Figure 2B). This tree showed the cocirculation of 2 genetically distinct lineages of H7 HA in European Mallards, 1 closely related to H7N7 and H7N1 HPAI strains causing outbreaks in the Netherlands (2003) and Italy (2000/2001) and 1 closely related to the H7N7 isolate obtained from a woman with conjunctivitis in the United Kingdom in 1996 (*20*). The maximum nucleotide/amino acid sequence identity between the Italian H7N1 HPAI virus A/Chicken/Italy/445/99 and the most closely related LPAI virus A/Mallard/Netherlands/12/00 was 98% nt and 98% amino acids (aa). The maximum nucleotide/amino acid identity between the Dutch H7N7 HPAI virus A/Chicken/Netherlands/1/03 and the most closely related LPAI virus A/Mallard/Netherlands/12/00 is 98% nt and 99% aa. The maximum nucleotide/amino acid identity between the LPAI virus A/Mallard/Sweden/56/02 and A/Turkey/Ireland/ PV74/95 (H7N7) was 95% nt, 96% aa; between A/Mallard/Sweden/56/02 and A/England/268/96 (H7N7), it was 96% nt and 97% aa.

We next analyzed the antigenic relatedness of the H7 influenza A viruses obtained from wild Mallards in HI assays with postinfection ferret antisera and hyperimmune rabbit antisera. The hyperimmune rabbit antisera were chosen on the basis of their ability to provide a broad response, which would recognize a wide range of strains within 1 subtype, whereas the postinfection ferret antisera were chosen on the basis of high specificity. HI assays showed that the antigenic properties of the H7 influenza A viruses from Mallards were relatively conserved, and that the HI data for the Mallard influenza A viruses did not differ significantly (i.e, up to 4-fold) from those obtained with strains causing the HPAI outbreak in the Netherlands in 2003 (Table 1). The antigenic analyses therefore confirmed the genetic data, which showed little genetic diversity between the H7 strains isolated from wild Mallards and the strains causing the H7 HPAI outbreaks.

**Table 1:** Hemagglutination inhibition assays with postinfection ferret antisera and hyperimmune rabbit antisera raised against H7 influenza A viruses

Virus	A/Eq/Prague/1/54*	A/Seal/Mass/1/80*	A/Neth/219/03†	A/Mallard/Neth/12/00†	A/Neth/33/03†
A/Equine/Prague/1/54 H7N7	**1:1,280‡**	1:1,280	<1:20	<1:20	<1:20
A/Seal/Massachusetts/1/80 H7N7	1:160	**1:1,280**	<1:20	<1:20	1:20
A/Netherlands/219/03 H7N7	1:160	1:1,280	**1:40**	1:40	1:80
A/Mallard/Netherlands/12/00 H7N3	1:160	1:1,280	1:20	**1:80**	1:40
A/Netherlands/33/03 H7N7	1:320	1:1,280	1:80	1:160	**1:160**
A/Mallard/Sweden/56/02 H7N7	1:640	1:5,120	1:80	1:80	1:160
A/Mallard/Sweden/105/02 H7N7	1:320	1:2,560	1:80	1:80	1:80
A/Mallard/Sweden/85/02 H7N7	1:160	1:1,280	1:40	1:80	1:80

*Hyperimmune rabbit antisera. †Postinfection ferret antisera. ‡Homologous titers are represented **boldfaced and underlined**.

### H5 Sequence Analysis, Phylogeny, and Antigenic Characterization

**Figure 3:**
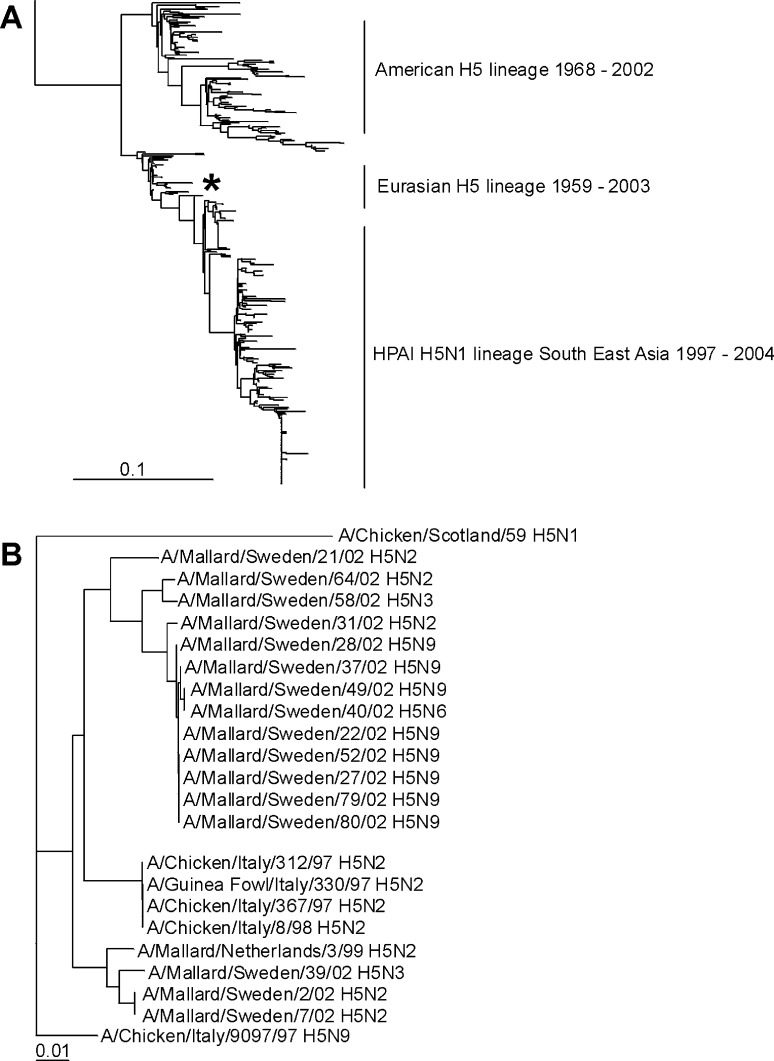
Phylogenetic trees of H5 sequences. A) Phylogenetic tree based on the amino acid sequence distance matrix, representing all H5 amino acid sequences available from public databases. The scale bar represents ≈10% of amino acid changes between close relatives. *Represent location of the H5 influenza A viruses isolated from Mallards. B) DNA maximum likelihood tree for the cluster of European H5 influenza A viruses and the low pathogenic avian influenza H5 influenza A viruses isolated from migrating Mallards by using A/Chicken/Scotland/59 as outgroup. The scale bar represents ≈1% of nucleotide changes between close relatives.

Sequence analyses of the HA ORFs of the 17 H5 influenza A viruses isolated from Mallards showed that the HA0 cleavage site lacked basic amino acid residues, which is typical for LPAI viruses. To determine the genetic relationship between the H5 influenza A virus isolates obtained from wild birds and strains causing recent H5 HPAI outbreaks (H5N2 in Italy 1997), we generated a phylogenetic tree based on the amino acid sequences of the HA1 domain of all H5 influenza A viruses currently available from public sequence databases (Figure 3A). As for H7, this tree showed the 2 clearly distinguishable Eurasian and American genetic lineages. The H5 HA sequences that we obtained from influenza A viruses isolated from European Mallards were closely related to the influenza A virus strains responsible for the H5 HPAI outbreak in Italy in 1997. The H5 HPAI influenza A strains isolated in Southeast Asia beginning in 1997 form a continuous genetic lineage, presumably evolving from a common LPAI wild bird ancestor around 1997 (*15*). Similarly, we did not detect close relatives of the recent HPAI Asian strains in Mallards in Europe (Figure 3A). The DNA maximum likelihood tree based on the full-length HA nucleotide sequences of the 17 H5 HA genes of Mallard influenza A viruses and those of the Italian H5 HPAI influenza A viruses confirmed the close genetic relationship (Figure 3B). Maximum nucleotide/amino acid identity between the Italian HPAI virus A/Chicken/Italy/312/97 H5N2 and the most closely related LPAI virus, A/Netherlands/3/99 H5N2, is 96% nt identity and 98% aa identity (Figure 3B).

In HI assays with postinfection ferret antisera and hyperimmune rabbit antisera raised against H5 influenza A viruses, the influenza A viruses obtained from wild Mallards were antigenically conserved and did not differ significantly (up to 4-fold) from the prototypic strains used in the HI assay (Table 2). In agreement with the phylogenetic analysis of the current H5N1 HPAI influenza A viruses from Southeast Asia, the antigenic properties of H5N1 influenza virus A/Vietnam/1194/04 differ significantly from those of the LPAI strains isolated from Mallards used in this study, when analyzed with the highly specific postinfection ferret antisera. Hyperimmune rabbit antisera failed to discriminate the antigenic properties of all strains because of the broader antigenic reactivity of these sera.

**Table 2:** Hemagglutination inhibition assays with postinfection ferret antisera and hyperimmune rabbit antisera raised against H5 influenza A viruses

Virus isolate	A/Tern/SA/61^1^	A/Tern/SA/61^2^	A/Dk/HK/205/77^1^	A/Dk/HK/205/77^2^	A/HK/156/97^1^	A/HK/156/97^2^
A/Tern/South Africa/61 H5N3	**1:640^‡^**	**1:320**	1:80	1:640	1:80	1:20
A/Duck/Hong Kong/205/77 H5N3	1:1,280	1:640	**1:240**	**1:1,280**	1:160	1:80
A/Hong Kong/156/97 H5N1	1:1,280	1:640	1:320	1:1,280	**1:640**	**1:320**
A/Vietnam/1194/04 H5N1	1:1,280	1:40	1:640	1:80	1:640	<1:20
A/Mallard/Sweden/21/02 H5N2	1:640	1:320	1:160	1:640	1:160	1:20
A/Mallard/Sweden/49/02 H5N9	1:320	1:320	1:40	1:320	1:160	1:40
A/Mallard/Netherlands/3/99 H5N2	1:640	1:1,280	1:160	1:5,120	1:160	1:80
A/Mallard/Sweden/7/02 H5N2	1:1,280	1:640	1:320	1:1,280	1:320	1:40

*Hyperimmune rabbit antisera. †Postinfection ferret antisera. ‡Homologous titers are represented **boldfaced and underlined**.

## Discussion

Because HPAI outbreaks in poultry find their origin in LPAI viruses present in waterfowl, influenza A virus surveillance in wild birds could function as an early warning system for HPAI outbreaks and as a means to keep panels of reference reagents, required for diagnostic purposes and vaccine production, up-to-date (*39**,**40*). Wild bird surveillance would also be relevant for HPAI viruses that represent pandemic threats. However, limited information on the prevalence of avian influenza A viruses in wild birds in Europe, and on the genetic and antigenic variability of the viruses in this part of the world, has made assessing the value of such surveillance studies difficult. We isolated avian influenza A viruses of subtypes H5 and H7 from Mallards in northern Europe. During a 4-year surveillance period, we isolated influenza A viruses of subtypes H5N2, H5N3, H5N6, H5N9, H7N3, H7N7, and H7N9, among many other influenza A virus isolates. All of these H5 and H7 influenza A virus isolates were obtained from Mallards during fall migration at a Swedish location and at 2 Dutch wintering sites. Using this relatively limited setting, we isolated influenza A viruses that possess H5 and H7 glycoproteins and gene segments closely related to those of influenza A viruses responsible for HPAI outbreaks in Europe, H5N2 in Italy (1997), H7N1 in Italy (1999–2000), and H7N7 in the Netherlands (2003). Thus, we conclude that influenza A virus surveillance in wild birds is useful to keep the panels of reference reagents up-to-date. Whether surveillance studies could be useful as a sentinel system is uncertain.

We observed minor antigenic and genetic diversity between the HA genes of Mallard influenza A virus isolates and those of HPAI virus strains. This finding implies that the influenza A virus isolates obtained during wild bird surveillance studies may also be prototypic vaccine candidates for human or veterinary use. Limited numbers of prototype vaccine strains, representing both the American and Eurasian genetic lineages of influenza A virus, could be generated to cover a wide range of HPAI strains. Such vaccine seed strains can be produced well ahead of outbreaks in poultry, other animals, or humans. The disadvantage of the minor antigenic differences between the vaccine strain and the epidemic strains will likely be compensated by the immediate availability of the vaccine. An additional advantage of the use of LPAI strains from wild birds as prototype vaccine strains is that they do not contain a basic cleavage site in the HA gene. Before HPAI strains can be used as vaccine candidates, the basic amino acid residues in the HA gene need to be removed by using reverse genetics technology; this would result in an extra modification step, which would consume precious time. Moreover, these vaccine strains can only be generated after an outbreak of HPAI has started.

We suggest that a thorough genetic and antigenic characterization of avian influenza A viruses isolated in the Americas, Asia, and Europe would be useful to prepare for outbreaks. While this usefulness has been demonstrated in our study with influenza A viruses of the H5 and H7 subtypes, it should be applied also to other influenza A virus strains relevant to animal and public health, in particular, those of subtypes H1, H2, H3, and H9.
